# Evaluation of mRNA Contents of *YBX2* and *JHDM2A* Genes on Testicular Tissues of Azoospermic Men with Different Classes of Spermatogenesis

**DOI:** 10.22074/cellj.2015.518

**Published:** 2015-04-08

**Authors:** Reza Najafipour, Sahar Moghbelinejad, Amir Samimi Hashjin, Farzad Rajaei, Zahra Rashvand

**Affiliations:** 1 Cellular and Molecular Research Centre, Qazvin University of Medical Sciences, Qazvin, Iran; 2 Department of Medical Genetics, Qazvin University of Medical Sciences, Qazvin, Iran

**Keywords:** Azoospermia, *YBX2*, *JHDM2A*

## Abstract

**Objective:**

Animal model studies have shown that *MSY2* and *JHDM2A* genes have an important role in spermatogenesis process and fertility of male mice. But the potential role of these genes in human spermatogenesis and fertility is not known yet. Therefore, we evaluated expression ratios of these genes in testis tissues of men with normal and impaired spermatogenesis.

**Materials and Methods:**

In this experimental study, after RNA extraction and cDNA syn- thesis from 50 non-obstructive azoospermic and 12 normal testis tissues, the expression ratios of genes were evaluated by real time polymerase chain reaction (PCR) technique. Hematoxcylin and eosin (H&E) staining was used for histological classification of testis tissues. For statistical analysis, one way analysis of variance (ANOVA) test was carried out.

**Results:**

Our results showed a significant reduction in mRNA level of *YBX2* in samples with impaired spermatogenesis (p<0.001) compared to samples with qualitatively normal spermatogenesis and normal spermatogenesis; however, in *JHDM2A* gene, despite sensible reduction in gene expression level in men with impaired spermatogenesis, no significant differences were shown (p>0.05). Furthermore in *YBX2*, a significant negative correlation was demonstrated between the efficiency score of spermatogenesis and the threshold cycle (CT) (r=-0.7, p<0.0001), whereas in *JHDM2A*, this negative correlation was not significant (r=-0.4, p=0.06).

**Conclusion:**

Generally, these data indicated that *YBX2* and *JHDM2A* genes may play an important role in male infertility, and suggested that these molecules can act as useful biomarkers for predicting male infertility.

## Introduction

*YBX2* and *JHDM2A* genes are germ-cell-specific molecules which are essential for the production of functional spermatozoa; therefore, their inactivation could lead to male infertility. Y box binding protein 2 ( *YBX2* ), also known as Contrin, is a human homologue of Xenopus DNA/RNA-binding and mouse MSY2 proteins (1,2). The gene encoding Contrin or *YBX2* is located on human chromosome 17p11.2-13.1. This gene appears to be testis-specific and distinct from other mammalian Y-box-binding proteins (3). In the mouse testis, this gene is expressed from pachytene spermatocyte to late spermatid stages, but the active form of *YBX2* factor is in round spermatid stage (4). YBX2 protein is highly similar to its mouse and xenopus germ-cell Y-box protein homologues. *YBX2* also acts as an mRNA stabilizer and a transcription factor of some testis specific genes such as *protamine genes*. Consequently, its loss of expression is likely to contribute to the nuclear condensation defects that occur in *MSY2*-null late-stage spermatids (4,6). Histone H3 lysine 9 ( H3K9 ) methylation is one of the best-characterized modifications in the study of germ cell development, such as meiotic chromosomal recombination, segregation, and histone removal followed by chromatin condensation in spermiogenesis. 

In order to do these processes, simultaneous function of methyltransferase and demethylase enzymes is unavoidable (7). JmjC-containing histone demethylase 2a ( *JHDM2A* ), also known as *JMJD1A* or *KDM3A,* has been identified as an H3K9 demethylase ( for monomethylation and dimethylation ), while it has been originally cloned as a testis-specific gene transcript (7). Location of this gene on human chromosome is 2p11.2. *JHDM2A* specifically regulates the expression of the genes encoding transition protein 1 ( Tp1 ) and protamine proteins, while this gene is necessary for the proper chromatin reorganization during spermatid maturation by directly promoting the transcription of the transition nuclear protein 1 ( *TNP1* ) and protamine1 ( *PRM 1* ) genes (8,9). Since both of two candidate genes in this research involve in expression regulation of some testis specific and important genes such as *PRM1, PRM2, TNP1, TNP2,* etc., different studies have pointed out that targeting *YBX2* and *JHDM2A* genes in animal models causes male infertility and different degrees of impaired spermatogenesis. To access this fact, for the first time, we evaluated the expression level of these genes in testicular tissues of men with non-obstructive azoospermia, with different class of spermatogenesis. 

## Materials and Methods

### Testicular tissue

This experimental study was approved by the Ethical Committee of the Faculty of Medical Sciences of Qazvin University of Medical Sciences, Qazvin, Iran. Sampling was done under supervision of urologist in Fertility and Infertility Center of Shariati Hospital., Tehran, Iran, during 2012. After patients gave an informed written consent, testicular biopsies were obtained from 50 azoospermic men with mean age of 31.3 ± 3.7 years old; these patients were candidates of assisted reproductive technique (ART) and exhibited impaired spermatogenesis. In 12 patients with obstructive azoospermia after vasectomy, biopsies were carried out for diagnostic reasons during vasectomy reversal. These biopsies revealing normal spermatogenesis were served as controls, while the mean age of these individuals was 35 ± 2.9 years old. The limitation of this study was scarcity of tissue samples with normal spermatogenesis, for this reason sampling time was prolonged.

To decrease possible confounding factors, the patients were excluded for these criteria: Y chromosome microdeletion, cystic fibrosis, varicocele, Klinefelter syndrome, or exposure to chemotherapy and radiation. In infertile patients with non-obstructive azoospermia, one part of testicular tissue specimens was used for testicular sperm extraction, while the other part was cut into two pieces; one piece was immediately prepared and frozen for RNA extraction procedure, and the other piece was fixed in Bouin’s fixative (Sigma, USA) and embedded in paraffin.

For histological evaluation, 5-μm-thick paraffin sections were stained in hematoxylin and eosin (H&E), and then scored according to the modified Johnsen scoring system. In this system of classification, all tubular sections in each piece of the testicular biopsy are evaluated systematically, and each is given a score from 1 to 10 as follows: score 10 including complete spermatogenesis with many spermatozoa; score 9 including slightly impaired spermatogenesis, many late spermatids, and disorganized epithelium; score 8 including less than five spermatozoa per tubule and few late spermatids; score 7 including no spermatozoa, no late spermatids and many early spermatids; score 6 including no spermatozoa, no late spermatids and few early spermatids; score 5 including no spermatozoa or spermatids and many spermatocytes; score 4 including no spermatozoa or spermatids and few spermatocytes; score 3 including only spermatogonia; score 2 including no germinal cells andSertoli cells only; as well as score 1 no seminiferous epithelium (10). To follow this classifying method, our samples were divided into 3 main groups based on above scoring: normal spermatogenesis (score 10), qualitatively normal spermatogenesis (scores 8-9), and impaired spermatogenesis (scores 1-7) (Table 1).

**Table 1:** Characterization of patient groups I-III and results of quantitative PCR analysis


Histological classification	Group 1	Group 2	Group 3
	Normal spermatogenesis	Qualitatively normal spermatogenesis	Impaired spermatogenesis

**Scores**	10	8-9	1-7
**Number of patients**	12	16	34
**Ct *YBX2* (mean ± SD)**	21.75 ± 1.2	21.2± 0.99	24.1 ± 1.9
**Ct GAPDH (mean ± SD)**	23.6 ± 0.85	23.1± 1.1	23.12 ± 2.3
**2^-ΔCt^**	3.6 ± 1.8	3.7 ± 1.5	0.5 ± 0.7
**Ct *JHDM2A* (mean ± SD)**	25.9 ± 0.96	26.33 ± 1.1	27 ± 1.7
**Ct GAPDH (mean ± SD)**	23.95 ± 1.5	24.3± 1.4	24.5 ± 1.2
**2^-ΔCt^**	0.25 ± 0.19	0.24± 0.15	0.17 ± 0.1


PCR: Polymerase chain reaction and SD; Standard deviation.

### RNA extraction and first strand cDNA synthesis

In summary, frozen testis tissues are homogenized in lysis buffer (Qiagen, Germany) using an ultrasonic processor UP100H (100W, 30 kHz) (Hielsher, Germany), and RNA is then extracted using RNeasy Mini Kit (Qiagen, Germany). In this regard, 1 volume of 70% ethanol was added to the homogenized lysate, and up to 700 μl of the sample was then transferred to an RNeasy spin column placed in a 2 ml collection tube. We used column DNase digestion to remove the residual DNA. After using different washing buffers, the RNeasy spin column was placed in a new 1.5 ml collection tube, 30-50 μl RNase-free water was added directly to the spin column membrane to elute the RNA, and finally RNA was frozen at -80˚C. Quality and quantity of isolated total RNA was measured using Nano Drop 2000c (Thermo, USA). In this case, RNA samples with A260/A280 ratios of >2 were selected for quantitative analysis. First strand complementary DNA (cDNA) synthesis was also performed using the RevertAid First Strand cDNA Synthesis Kit (Thermo Scientific, Fermentas, Waltham, MA, USA). For cDNA synthesis, 1-5 μg total RNA and 0.5 μg oligo (dt) were added to sterile, nuclease-free tube on ice, and total volume reached to 11.5 μl with DEPC-treated water. This solution was mixed and centrifuged briefly and then was incubated at 65˚C for 5 minutes. Next 4 μl 5X reaction buffer for reverse transcription, 0.5 μl RiboLock™ RNase inhibitor, 2 μl dNTP mix and 1 μl revertaid™ H minus reverse transcriptase (Bioneer, Korea) were added to solution, and total volume reached to 25 μl with DEPC-treated water. After being centrifuged, the solution was incubated for 60 minutes at 42˚C. Ultimately the reaction was terminated by heating at 70˚C for 10 minutes.

### Real-time polymerase chain reaction (PCR) and comparative threshold cycle (CT) method

In this experiment, we used GAPDH gene as an internal control for quantification of target genes expression. Two target genes (*YBX2* and *JHDM2A*) and GAPDH (as internal control) were amplified with appropriate primers and probes (Table 2). All primers and probes were designed using gene runner software version 3.05 (Hastings SoftwareInc. Hastings, NY, USA). TaqMan real time PCR assay was carried out in final reaction volumes of 20 μl with 10 μl of a TaqManMaster Mix (Takara, Shiga, Japan), 0.2 μM of forward and reverse primers, and 2 μl of cDNA. Thermal cycling was performed using the ABI-7500 sequence detection system (Applied Biosystems, Foster, CA, USA) with the following cycling condition: 30 seconds at 95˚C as first denaturation step, followed by 40 cycles at 95˚C for 5 seconds and 60˚C for 34 seconds.

**Table 2:** The oligonucleotid primers and probes used in real time PCR assay


Target and internal (bp) control genes	Sequence	Amplicon size

***YBX2***	F: CCCTACCCAGTACCCTGCT	150
	R: CCTTCCTTCAACCCTTGATAA	
	P: CAGGAGGACCAAAGCAGCAGCC	
***JHDM2A***	F: GTTCCACAAGCATTGACTGG	145
	R: CTGGTGCATTTGAAACATCC	
	P: TGCCAATCCTCCTGAACTGCAGA	
**GAPDH**	F: TCAAGAAGGTGGTGAAGCAG	93
	R: CGCTGTTGAAGTCAGAGGAG	
	P: CCTCAAGGGCATCCTGGGCT	


Optimization of real-time PCR assay

### Optimization of real-time PCR assay

The efficiency of each gene was determined using 10 fold series of normal cDNA dilutions. Validation of experiments was performed to determine PCR efficiencies of target and reference gene. The slopes of the fit lines were within the acceptable range of -3.6<slope<-3.1. PCR efficiencies of target genes and reference gene were approximately equal, indicating validity of the comparative threshold cycle method for quantifying the expression. Each assay was repeated at least two times before drawing any conclusions. The 2^−ΔCT^ method of relative quantification was used to determine the fold change in expression. This was done by normalizing the resulting CT values of the target mRNAs to the CT values of the internal control () in treated and untreated samples (ΔCT=CT_target_–CT_GAPDH_) (11).

### Statistical analysis

Statistical analysis including mean, standard deviation (SD), and correlation coefficients (R) were done using Prism (version 3) software (GraphPad Prism, California, USA).

Additionally, one-way analysis of variance (ANOVA) was carried out to determine the significant differences between the studied groups. A p value of <0.05 was considered as statistically significant.

## Results

**Fig.1:**
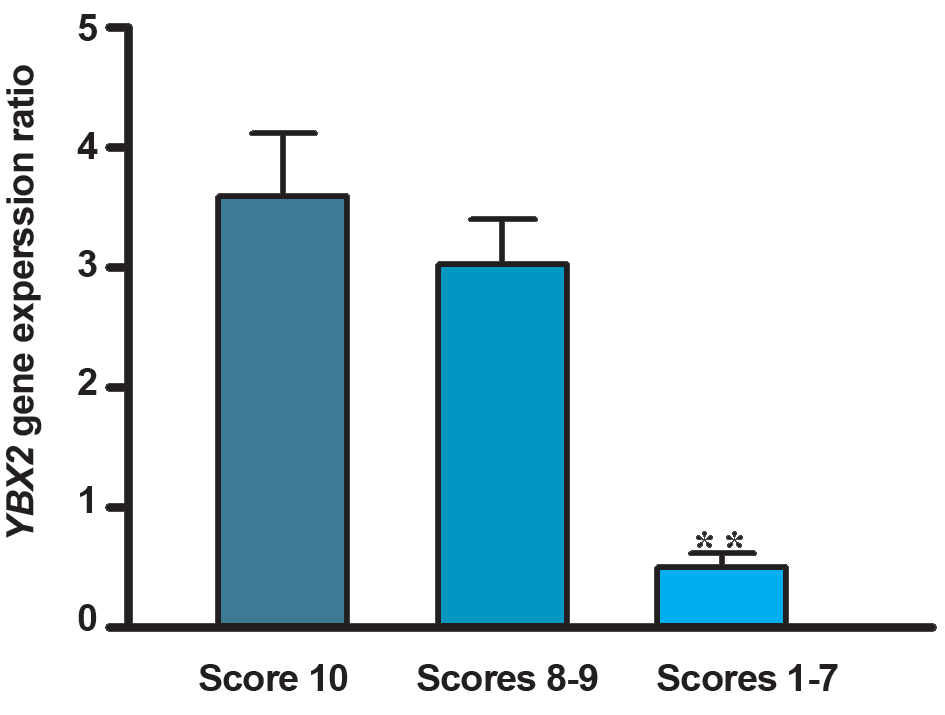
mRNA expression of YBX2. Men with normal spermatogenesis ( n=12, score 10 ), men with qualitatively normal spermatogenesis ( n=16, scores 8-9 ) and in azoospermic men with impaired spermatogenesis ( n=34, scores 1-7 ). Values are presented as mean ± SD. **; P<0.001 as compared to control groups using one way ANOVA test. SD; Standard deviation and ANOVA; One-way analysis of variance.

**Fig.2:**
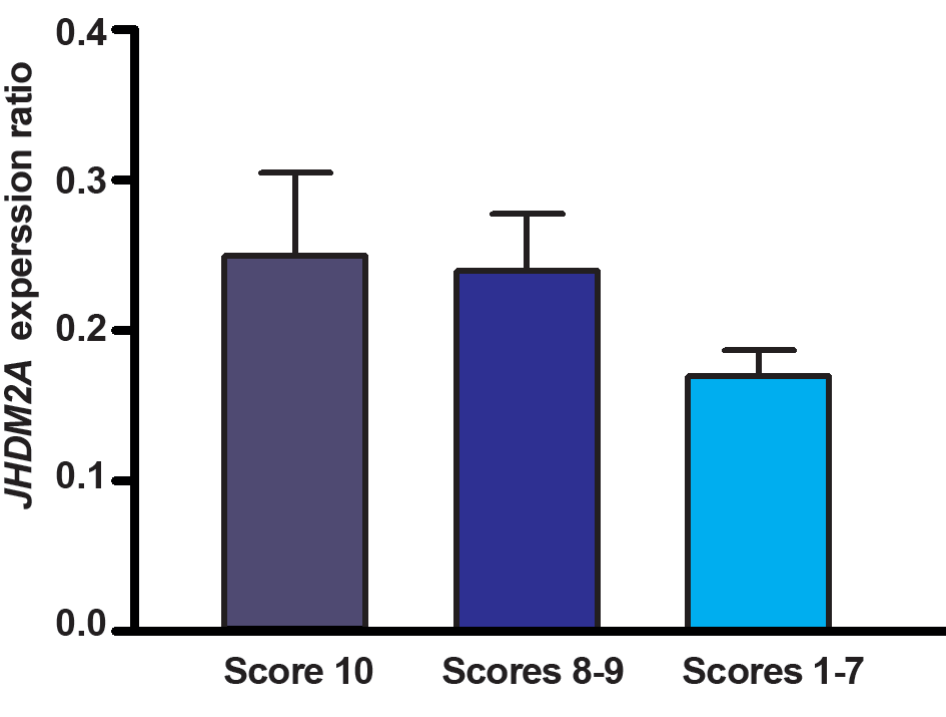
mRNA expression of JHDM2A. Men with normal spermatogenesis ( n=12, score 10 ), men with qualitatively normal spermatogenesis ( n=16, scores 8-9 ), and in azoospermic men with impaired spermatogenesis ( n=34, scores 1-7 ). Values are presented as mean ± SD. SD; Standard deviation.

**Fig.3:**
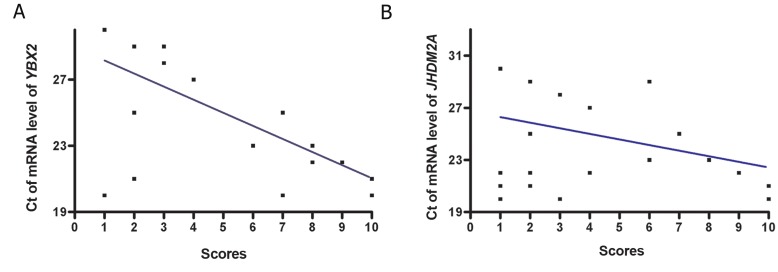
A. Scatter plots of CT of mRNA level of YBX2 (r=-0.7; p<0.001) and B. CT of mRNA level of JHDM2A (r=-0.4; p= 0.06). mRNA against score value for efficiency of spermatogenesis with regression lines. CT; Threshold cycle.

Men with impaired spermatogenesis (group III) exhibited a significant (p<0.001) lower mRNA level of *YBX2* (2^-ΔCt^: 0.5 ± 0.7) when compared to the samples with normal spermatogenesis (group I) (2^-ΔCt^: 3.6 ± 1.8) and qualitatively normal spermatogenesis (group II) (2^-ΔCt^: 3.03 ± 1.5). In the testicular tissues of azoospermic men with impaired spermatogenesis, *YBX2* transcripts were 7.2 folds less than testicular tissues of men with normal spermatogenesis (Fig.1), but there is no significant difference in mRNA ratio (2^-ΔCt^) of *YBX2* between the samples of group I and group II (p>0.05). The mRNA content of *JHDM2A* in testis tissues of studied groups showed that despite sensible reduction in gene expression in samples with impaired spermatogenesis (2^-ΔCt^: 0.17 ± 0.1), compared to samples with normal and qualitatively normal spermatogenesis (2^-ΔCt^: 0.25 ± 0.19 and 2^-ΔCt^: 0.24 ± 0.15, respectively), there was no significant difference in 2^-ΔCt^ ratio among the three studied groups (p>0.05, Fig.2). A significant negative correlation could be seen between the efficiency score of spermatogenesis and the mean CT value of mRNA level of *YBX2* (r=-0.7, p<0.0001), whereas in *JHDM2A*, this negative correlation was not significant (r=-0.4, p=0.06, Fig.3A, B).

**Fig.4:**
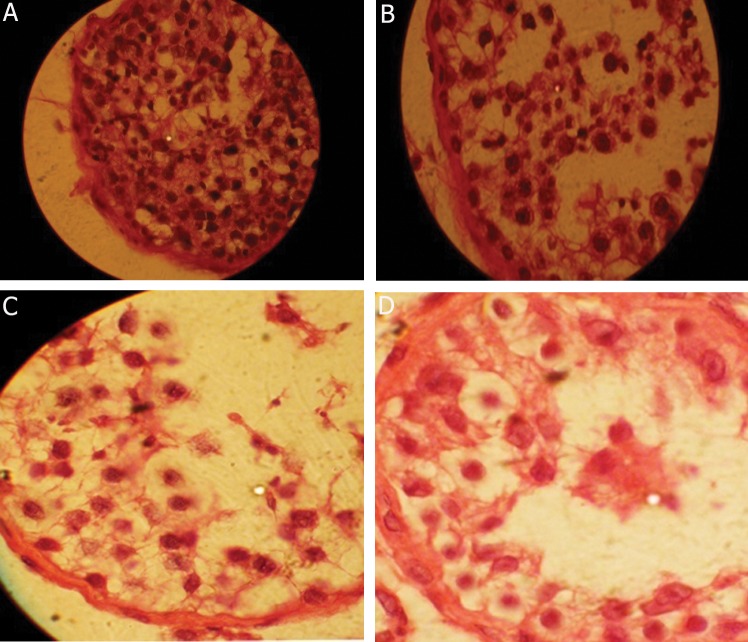
Typical photomicrographs of hematoxcylin and eosin (H&E) staining of tissues, magnification ×1000. A. Hyospermatogenesis, B. Maturation arrest in round spermatid stage, C. Maturation arrest in spermatocyt stage and D. Maturation arrest in spermatogony stage.

Generally, a negative correlation could be seen between the efficiency of spermatogenesis in different studied groups, in a way that down regulation of *YBX2* was seen significantly in comparison to normal ones in groups with different levels of impaired spermatogenesis (Fig.4).

## Discussion

Generally 5% of adult human males are affected by male infertility, whereas, 75% are diagnosed as idiopathic due to unknown molecular mechanisms underlying the defects (12). Spermatogenic cells are known through regulated spatiotemporal gene expression and strongly repressed translation in meiotic and haploid male germ cells. A vast variety of molecules and proteins are involved in these processes (13,14). In this research, we investigated two of these important molecules ( YBX2 and *JHDM2A* ). The proteins encoded by these two genes express specifically and highly in testicular tissue and are involved in functional regulation of some strategicmeiotic and post meiotic genes. Contrin ( *YBX2* ) is a transcription and translation regulatory factor and a germ-cell-specific molecule which is essential for the production of functional spermatozoa. 

About the molecular function of this protein, animal models and *in vitro* assay studies showed that *MSY2* acts as a transcription factor and an mRNA stabilizer which regulates expression of some testis specific genes in transcription and translation level. In this regard, *MSY2* marks specific mRNAs ( those transcribed from Y-box promoters ) in the nucleus for cytoplasmic storage, thereby links mRNA transcription and storage/translational delay. In this process, *MSY2* recognizes the CTG ATTGGC/ TC/TAA sequence as a DNA motif in the promoter of many genes that are specifically expressed in male germ cells. After *MSY2* binds to its consensus promoter sequence, binds to transcripts of this gene. Subsequently this protein stabilizes and represses their translation as RNAprotein complexes (15,17). 

Yang et al. (18) generated *MSY2*-null mice. They found that the mutant males had an abnormally high number of apoptotic meiotic spermatocytes, lacked spermatozoa in the epididymis, and were sterile. Their results emphasized on the major role of this protein in male fertility. Meng et al. (19) and Deng et al. (20) reported single nucleotide polymorphisms ( SNP ) of this gene in China’s population, while SNP frequency was significantly higher in infertile men compared to fertile ones. In other study, Hammoud et al. (21) investigated *YBX2* gene alterations in blood samples of men with severe defects in spermatogenesis, including azoospermia, severe oligozoospermia, and abnormal protamine expression. Their results showed seven polymorphisms were present at a statistically higher frequency in patients with infertility, particularly in men with abnormal protamine expression. This study showed t hat *YBX2* gene has a potential role in male spermatogenesis, leading to male factor infertility (21). Our results, in the same path, showed significant down-regulation of this gene in testicular tissues of azoospermic men with impaired spermatogenesis compared to men with normal spermatogenesis ( p<0.001 ) (Fig.1A,Table 2). There is asignificant negative correlation between the score for efficiency of spermatogenesis and the mean CT value of mRNA level of *YBX2* in our samples, indicating the role of this gene in human spermatogenesis. Other regulatory factor which was studied in this research was *JHDM2A* gene. Okada et al. (22) originally cloned this gene as a testis-specific gene transcript. Their results showed an intense nuclear expression of this gene in round spermatids and a sub-nuclear distribution. Co-expression of *JHDM2A* gene with RNA polymerase II indicates that this gene may contribute to transcriptional activation of some testis specific genes. *JHDM2A* also stimulates *TNP1* and *PRM 1* genes, by bonding to core promoter and removing H3K9 methylation. Histone demethylase *JHDM2A* is critical for *TNP1* and *PRM 1* transcription and spermatogenesis (22). *Jhdm2a*-deficient mice have shown infertility and smaller testes (23). Despite the fact that animal model studies showed the role of this protein in male infertility, expression ratio of this gene did not show significant difference between azoospermic and fertile men samples ( p>0.05 ). Despite the negative correlation between the efficiency score of spermatogenesis and the mean CT value of mRNA level of *JHDM2A* in studied samples, this correlation was not significant. Probably the role of this gene is not very influential in human fertility; therefore, more samples must be studied. 

## Conclusion

Our findings indicated significant down-regulation of *YBX2* gene in testis tissue of azoospermic men, but future studies are needed to investigate the role of *YBX2* and *JHDM2A* genes in regulating some specific genes of testis involving in human meiotic and post meiotic. 
